# A Perfect Storm: The Convergence of Aging, Human Immunodeficiency Virus Infection, and Inflammasome Dysregulation

**DOI:** 10.3390/cimb46050287

**Published:** 2024-05-15

**Authors:** Siva Thirugnanam, Namita Rout

**Affiliations:** 1 Division of Microbiology, Tulane National Primate Research Center, Covington, LA 70433, USA; thiru@tulane.edu; 2 Department of Microbiology and Immunology, Tulane University School of Medicine, New Orleans, LA 70112, USA; 3 Tulane Center for Aging, Tulane University School of Medicine, New Orleans, LA 70112, USA

**Keywords:** aging, HIV, inflammasome, inflammaging, cell signaling

## Abstract

The emergence of combination antiretroviral therapy (cART) has greatly transformed the life expectancy of people living with HIV (PWH). Today, over 76% of the individuals with HIV have access to this life-saving therapy. However, this progress has come with a new challenge: an increase in age-related non-AIDS conditions among patients with HIV. These conditions manifest earlier in PWH than in uninfected individuals, accelerating the aging process. Like PWH, the uninfected aging population experiences immunosenescence marked by an increased proinflammatory environment. This phenomenon is linked to chronic inflammation, driven in part by cellular structures called inflammasomes. Inflammatory signaling pathways activated by HIV-1 infection play a key role in inflammasome formation, suggesting a crucial link between HIV and a chronic inflammatory state. This review outlines the inflammatory processes triggered by HIV-1 infection and aging, with a focus on the inflammasomes. This review also explores current research regarding inflammasomes and potential strategies for targeting inflammasomes to mitigate inflammation. Further research on inflammasome signaling presents a unique opportunity to develop targeted interventions and innovative therapeutic modalities for combating HIV and aging-associated inflammatory processes.

## 1. Introduction

According to WHO reports, since the beginning of the human immunodeficiency virus (HIV) epidemic 40 years ago, around 85.6 million individuals have been affected, resulting in an estimated 40.4 million lives lost due to HIV-related causes. At present, approximately 40 million people worldwide are estimated to be living with HIV infection, comprising roughly 20 million men, 17.4 million women, and 1.5 million children under 15. While sub-Saharan Africa has the most people living with HIV, new infections are rising faster in Europe, South America, North America, and other regions [1]. According to the CDC, white individuals in the United States have HIV incidence rates (new infections per 100,000 population) that are 7.8 times lower than those of Black/African American persons and 3.9 times lower than those of Hispanic/Latino persons. Consequently, HIV prevalence among Black/African American individuals is seven times higher than among white individuals. Low-income countries still bear the primary burden of HIV/AIDS globally, with individuals living with HIV in these regions facing challenges in accessing healthcare services, such as timely HIV testing and consistent ART treatment [2]. The administration of combination antiviral therapy (cART), which involves treating patients with a combination of drugs, is an effective method for suppressing HIV replication [3]. Continuous cART has been shown to lead to the durable suppression of viral replication, likely providing lifelong effectiveness [4]. The early diagnosis and initiation of cART can bring the life expectancy of patients close to that of the general population [5]. Due to cART’s effectiveness in extending life expectancy, over half of the people with HIV (PWH) in the US are now over 50, with this number projected to reach 70% by 2030 [6]. Despite this, overall health differences between individuals with HIV and those uninfected persist, as individuals with HIV have a higher-than-expected risk of health problems including metabolic, cardiovascular, and neurological diseases [7,8]. Women, particularly PWH face a greater burden of age-related health problems compared to men, with the types of these issues also differing by sex [9]. The higher rates of co-morbidities in individuals with HIV are caused by several factors: the lingering effects of HIV infection itself, the complex side effects of ART drugs, the increased risk of opportunistic infections, and living longer with HIV. The presence of age-related comorbidities in PWH has led to the hypothesis that patients treated with ART may experience accelerated aging [10,11,12].

Although the mechanisms underlying age-related health issues in both the aging population and PWH are not yet clearly understood, it is well established that a persistent inflammatory state, driven by ongoing immune activation, is a major contributing factor to premature immunosenescence and accelerated aging observed in untreated PWH [10,13,14]. Studies in PWH indicate an increase in biological age of approximately five years in blood cells and seven years in the brain compared to uninfected individuals [15]. Similarly, aging populations are impacted by the rise in systemic inflammation, referred to as inflammaging, which occurs even in the absence of overt infection, and serves as a risk factor for age-related morbidity and mortality [16,17]. Furthermore, studies in centenarians suggest that managing chronic low-grade inflammation through a balanced regulation of inflammatory and anti-inflammatory responses might be crucial in promoting longevity and overall health [18,19]. The healthy aging observed in centenarians is attributed to a specific genetic characteristic that regulates inflammatory pathways differently from other elderly individuals affected by various age-related illnesses [19]. Overall, these studies support the concept that similar mechanisms of chronic inflammatory pathways are triggered by HIV infection and aging. Elevated levels of inflammasome signaling have been documented alongside chronic inflammation in both elderly individuals and PWH. However, in the field of inflammasome research, existing reviews have mostly examined the roles of inflammasomes in HIV infection and aging separately, shedding light on their individual impacts and implications. This review aims to bridge this gap by providing a comprehensive analysis of the interplay between inflammasomes, aging, and HIV infection, with a particular focus on the elderly population affected by HIV and PWH undergoing accelerated aging. Through our analysis, we seek to provide valuable insights that inform the development of novel therapeutic interventions and improve the overall well-being of elderly individuals living with HIV and those experiencing accelerated aging. By delving into the complex interactions between inflammasomes, aging, and HIV pathogenesis, we aim to provide valuable insights that will inform future research and ultimately lead to improved health outcomes for this vulnerable population.

## 2. HIV-Associated Chronic Inflammation

After entering the human body, HIV infects cells by utilizing CD4 and CCR5/CXCR4 as primary and secondary receptors, respectively, leading to infection in its primary target cells such as T cells, macrophages, and dendritic cells, thereby establishing a long-term infection. As viral replication and dissemination reach peak levels, it leads to the depletion of CD4+ T lymphocytes from the blood, lymphoid organs, and mucosal tissues [20]. The gastrointestinal (GI) tract, home to a substantial number of T lymphocytes, experiences a disproportionately higher depletion of CD4+ T-cells compared to peripheral blood [21]. Chronic HIV infection-driven immune activation, characterized by the elevated levels of HIV-specific CD8+ T cells, B cells, NK cells, pDCs, monocytes, and proinflammatory cytokines, happens in parallel with CD4+ T cell depletion in HIV infection [22,23]. The disruption of the GI tract’s immune system compromises the mucosal barrier, facilitating the translocation of microbial products into the bloodstream, which in turn is associated with chronic inflammation and sustained immune activation. Furthermore, alcohol abuse among people living with HIV exacerbates the disruption of the microbiota composition and compromises the integrity of the intestinal barrier, leading to elevated levels of microbial translocation and chronic immune activation [24].

Upon entry into the host cell, HIV is recognized by DNA sensors such as the endoplasmic reticulum adaptor stimulator of interferon genes (STING) in the cytosol, and it triggers the expression of IFN-α and IFN-β [25,26]. HIV sensing and viral replication involves cyclic GMP–AMP synthase (cGAS) and interferon γ-inducible protein 16 (IFI16) in macrophages and their expressions are elevated during the viral replication [26,27,28]. These cytoplasmic DNA sensors also recognize the reverse transcription products such as cDNA, ssDNA, DNA/RNA hybrids, and dsDNA [27,29]. The capsid proteins of HIV serve as target molecules for innate immune factors, including Tripartite Motif-containing protein 5 (TRIM5) and NONO [30,31]. Following the breach of initial defenses and viral replication, innate immune cells including NK cells, NKT cells, γδ T cells, dendritic cells, and macrophages respond to newly produced viral particles, triggering the activation of CD4+ and CD8+ T lymphocytes [32].

The hyperactive immune state emerges with the elevated proliferation of both specific and non-specific T cells alongside the increased production of proinflammatory cytokines [33]. The rapid turnover of CD4+ T cells provides numerous targets for HIV, leading to a surge in HIV viral loads. The elevated levels of HIV DNA and protein activate toll-like receptors (TLRs) and caspase-1, resulting in the release of proinflammatory cytokines and chemokines including type I IFNs, IL-6, TGFβ, IL-8, IL-1α, IL-1β, MIP-1α, MIP-1β, and RANTES into circulation [34,35]. GI mucosa disruption and microbial translocation further fuel the production of proinflammatory cytokines and immune activation. Microbial products like peptidoglycan, lipoteichoic acid, lipopolysaccharide (LPS), flagellin, and DNA engage with TLRs and nucleotide-binding oligomerization domains (NODs) to trigger robust proinflammatory responses [36]. Soluble CD14 (sCD14) which is a marker of LPS-induced monocyte or macrophage activation has a positive relationship with the high levels of LPS in PWH [37]. The persistent elevation of sCD14 and proinflammatory effector memory T cells reported in PWH implicates the synergistic role of aging and HIV infection in sustained low-grade inflammation, as well as gut barrier damage and permeability [38].

Unhealthy lifestyles among PWH exacerbate inflammation. Poor diet, alcohol consumption, smoking, and the lack of exercise can contribute to increased inflammation and the risk of various morbidities. These factors also elevate the risk of heart disease and hypertension among PWH [39]. Among PWH, engaging in injection drug use heightens the likelihood of progressing to AIDS, experiencing elevated immune activation, and facing increased mortality rates due to AIDS-related complications, comorbidities, and other infections, even with adherence to cART [40]. Substance abuse facilitates HIV replication and the infection of new cells, and amplifies bacterial translocation in PWH [41,42]. HIV infection and chronic opioid use are individually linked to systemic inflammation. However, chronic opioid use exacerbates systemic inflammation among PWH, with those who are HIV-positive and use opioids exhibiting the highest levels of systemic inflammation [43]. Several studies showed opioid exposure exacerbates the release of proinflammatory cytokines and chemokines induced by both HIV-1 Tat protein and gp120 envelope protein [44,45].

The chronic immune activation and inflammation caused by HIV infection remain unresolved despite achieving viral suppression with cART [46,47]. This is attributed to the failure of cART to completely restore gut barrier dysfunction despite effective viral suppression to undetectable levels in the blood [48,49,50]. Immune activation and inflammatory biomarkers are still significantly elevated in ART-treated and untreated patients with HIV than those in individuals without HIV [51,52,53,54]. Even amongst individuals effectively treated with ART for HIV, the persistence of low-grade chronic inflammation emerges as a significant contributor to the development of various age-related diseases, potentially impacting both morbidity and mortality, mirroring trends observed in the wider elderly population.

## 3. Inflammation of Aging

While several factors contribute to aging and associated comorbidities, a weakened immune environment, chronic inflammation, and compromised immune regulation are major contributors. These changes in the aging immune system are collectively termed as immunosenescence, which contributes to inflammaging, characterized by the elevated levels of inflammatory markers in both cellular and tissue compartments [55]. The process of immunosenescence promotes chronic inflammation, triggering the release of associated molecular patterns that further exacerbate immune senescence, thereby establishing a detrimental positive feedback loop. Senescent hematopoietic stem cells (HSCs) are attributed to imbalanced myelopoiesis and lymphopoiesis, leading to the release of proinflammatory cytokines such as IL-1, IL-3, IL-6, TNF-α, interferons, and GM-CSF. These cytokines and growth factors, in turn, promote HSC myeloid differentiation bias [56,57,58]. In aging mice, HSC exhibits a diminished capacity for self-renewal but in higher numbers which may be associated with the elevated levels of IFN-γ [59,60,61]. Exposure to microbial products like LPS, which triggers a proinflammatory environment, has been shown to impair HSC self-renewal and competitive repopulation activity [62].

Furthermore, increased microbial translocation and systemic inflammation in older vervet monkeys with poorer intestinal barrier function mimic the effects of HIV infection [63,64]. In accordance with the endotoxemic burden, innate immune responses, measured as circulating secretory IgA and antimicrobial peptide α-defensin 5, were both greater in old monkeys [64]. An analysis of colonic tissue from older baboons revealed a decreased expression of tight junction proteins zonula occluden-1, occludin, and junctional adhesion molecule-A, while claudin-2 expression increased. Colonic biopsies from older baboons displayed upregulated miR-29a and inflammatory cytokines IFN-γ, IL-6, and IL-1β compared to biopsies from young animals [65]. Additionally, a significant age-related increase in human and rhesus monkey plasma IL-6 and IL-1β, even in the absence of inflammation or infection, indicates that proinflammatory cytokines IL-6 and IL-1β are important mediators of the age-associated increase in intestinal permeability [66,67]. These studies suggest that an increased colonic permeability, caused by changes in intestinal epithelial tight junction proteins, might be a major factor contributing to inflammation in elderly individuals.

The aging process exerts a multifaceted influence on the immune system, impacting both its adaptive and innate arms. Antigen presentation by dendritic cells to CD4+ T cells is severely impaired and proinflammatory cytokines negatively influence the reactivity of the CD4+ T cells in aging. Aging CD4+ T-cells show several distinctive characteristics including the loss of proliferative capacity, low production of IL-2, and a loss of the expression of CD28 molecule [68]. CD4+ T cells in aged mice produce elevated levels of IL-17 compared to young populations [69], which is associated with several inflammatory conditions [70,71,72]. IL-17-producing γδ17 T cells dominate the γδ T-cell pool of aged mice mediated by increased IL-7 expression in the T-lymphocytes of old mice [73]. Aged skin exhibits an increase in IL-17-producing γδ T cells and innate lymphoid cells (ILCs), while blocking IL-17 signaling during this process attenuates the proinflammatory state and delays the onset of age-related characteristics [74].

## 4. Inflammaging and HIV Infection

**Figure 1:**
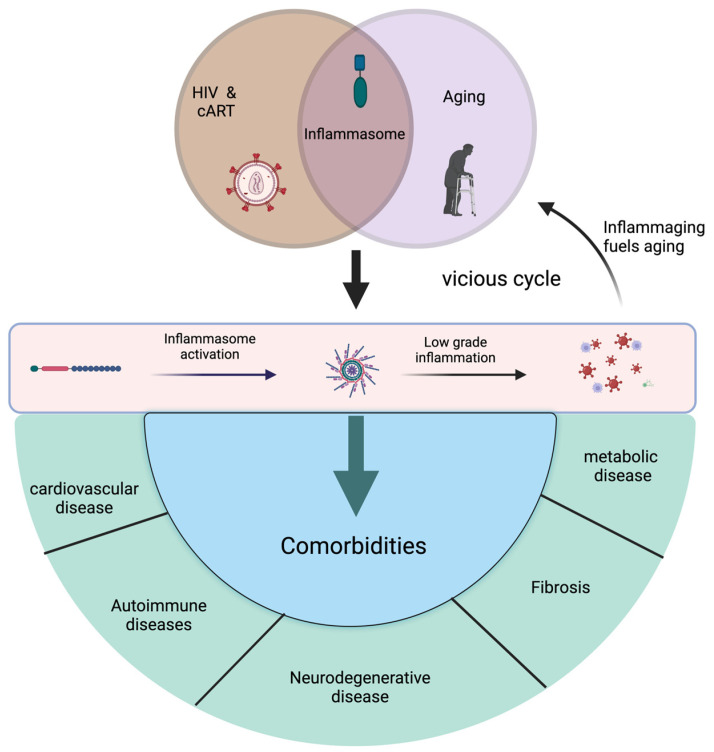
HIV infection and treatment, as well as aging, drive inflammasome activation that is associated with chronic immune activation and inflammation driven in part by inflammasome-mediated cytokine production. This low-grade chronic inflammation accelerates biological aging and increases vulnerability to comorbidities (shown in green) in both people with HIV and the elderly.

As observed in aged individuals, HIV infection exhibits progressive immune function decline and the long-term disruption of immune homeostasis, mirroring immunosenescence typically seen with aging. Immunosenescence is characterized, in part, by inflammaging, and is considered a key contributor to age-related morbidity and mortality also exhibited by PWH [75]. Studies in nonhuman primates demonstrate that natural SIV hosts, including sooty mangabeys and African green monkeys, exhibit mechanisms regulating chronic inflammation. This immune control translates to the absence of microbial translocation, immune activation, exhaustion, AIDS development, and accelerated aging observed in non-natural hosts [76]. While persistent chronic inflammation is linked to HIV infection and aging, the intracellular mechanisms responsible for driving this process, however, are largely unknown. Elucidating and targeting these pathways holds promise in mitigating inflammation and immune activation markers, potentially leading to a reduced risk of comorbidities in HIV infection and the aging population. The inflammasome pathway of the innate immune system is one of the major contributors to inflammation, initiating a proinflammatory response to HIV infection and during aging (Figure 1).

## 5. Inflammasomes

Inflammasomes are cytosolic multiprotein oligomers, functioning as key sensors within the innate immune system. Inflammasome protein machinery is expressed in macrophages, dendritic cells (DCs), neutrophils, and epithelial cells [77]. Upon the recognition of pathogen-associated molecular patterns (PAMPs) or damage-associated molecular patterns (DAMPs) within the cell cytoplasm, these complexes initiate and orchestrate a potent innate immune response. The recognition of inflammatory ligands by inflammasomes leads to the release of proinflammatory cytokines, which recruit and activate cells of the innate and adaptive immune system at the site of infection or stress.

Cytoplasmic pattern recognition receptors (PRRs), including nucleotide-binding oligomerization domain (NOD); leucine-rich repeat (LRR)-containing protein (NLR) family members NLRP1, NLRP3, and NLRC4; and absent in melanoma 2 (AIM2) and pyrin proteins can be involved in the assembly of inflammasomes [78]. The major classes of the sensory family include nucleotide-binding domain (NBD) and leucine-rich repeat (LRR)-containing proteins (NLRs) as well as the PYHIN protein family [78]. Based on N-terminal domain architecture, the NLR family is further classified based on recognizable N-terminal domains, including the acidic transactivation domain, pyrin domain, caspase recruitment domain (CARD), and baculoviral inhibitory repeat (BIR)-like domains [79]. The PYHIN protein family, which includes absent in melanoma 2 (AIM2) and interferon (IFN)-inducible factors, consists of HIN200 and pyrin domains but lacks NLR sensors [80].

Following activation, NOD-like receptors (NLRs) and other PYRIN domain (PYD)-containing proteins initiate the assembly of higher-order inflammasome complexes. These supramolecular signaling platforms comprise at least a sensor NLR and a caspase, and frequently incorporate the adaptor protein apoptosis-associated speck-like protein containing a CARD (ASC) [81,82]. The recognition of exogenous ligands, such as double-stranded DNA (dsDNA) and specific bacterial/viral proteins, by inflammasome PRRs triggers oligomerization for complex assembly. This process involves either the homo-oligomerization of the CARD domain or ASC. This assembly platform facilitates caspase-1 activation, leading to the proteolytic processing and the maturation of proinflammatory cytokines interleukin-1β (IL-1β) and interleukin-18 (IL-18) and additionally triggers the secretion of other proteins like IL-1α and fibroblast growth factor-2 (FGF-2) through an unconventional protein secretion pathway [83]. While NLRP3, AIM2, and others represent the canonical inflammasome pathway leading to caspase-1 activation and IL-1β/IL-18 maturation, receptors like NLRP3, NLRP6, NLRP12, and IFI16 exhibit functional diversity beyond caspase-1 activation. These receptors can also engage other cellular pathways, such as NF-κB, MAP3K, and STING signaling, highlighting their role in regulating inflammatory responses outside the canonical inflammasome pathway, known as the non-canonical inflammasome pathway [84,85,86].

The activation of inflammasomes leads to the release of mature proinflammatory cytokines triggered by caspase-1, as well as the induction of pyroptosis and caspase-8-driven apoptosis. The proteolytic cleavage of pro-IL-1β by caspases results in the rapid secretion of IL-1β from the cells [87]. The secreted IL-1β then induces the expression of proinflammatory genes such as human β-defensin-2 (HBD-2), TNF-α, IL-6, and GM-CSF [88]. Another cytokine, IL-18, processed by caspase-1, induces the production of IFN-γ and IL-17, along with other proinflammatory cytokines and chemokines, and activates IL-18 receptor alpha chain abundant γδ T cells to produce IL-17 [89,90]. Besides facilitating the maturation of IL-1β and IL-18, the initiation of an inflammatory form of cell death known as pyroptosis is another significant physiological consequence of caspase activation [91]. Canonical inflammasome complex employ caspase-1 while noncanonical inflammasome pathways involve caspase-4, caspase-5 (in humans), caspase-8, and caspase-11 (in mice) to cleave gasdermin D along with pro-IL-1β and pro-IL-18 [92,93]. The caspase-mediated cleavage of gasdermin D results in the generation of an N-terminal pore-forming domain, which oligomerizes and forms non-selective pores in the plasma membrane. This process leads to cell swelling, pyroptosis, and potentially cytokine release [93,94]. In addition to pyroptosis, the activation of inflammasomes is also involved in other forms of cell death, including apoptosis, necroptosis, and ferroptosis [95]. Caspase-8 serves as a pivotal regulator of cell death, playing a role in apoptosis, necroptosis, or pyroptosis depending on its post-translational modifications and the specific cell type [96]. Ferroptosis, an iron-dependent programmed cell death process, is influenced by proteins involved in pyroptosis pathways, with blocking gasdermin D suppressing both forms of cell death [96,97]. Thus, as inflammasome activation is closely linked to cell death pathways, the tight control of inflammasome activity is essential for a balanced immune system functioning. Conversely, uncontrolled inflammasome activation can trigger unrestrained inflammatory responses, potentially paving the way for a spectrum of inflammatory disorders.

### 5.1. Inflammasomes and HIV

**Figure 2:**
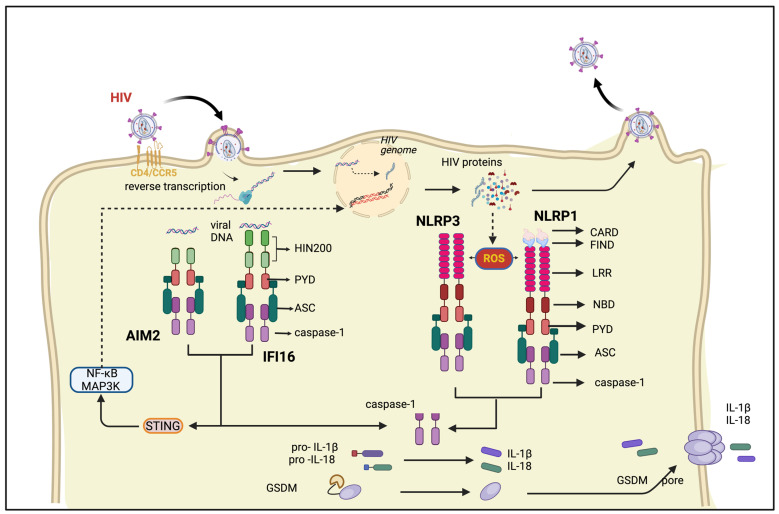
HIV infection initiates the inflammasome pathway by interacting with CD4 and coreceptors like CCR5, facilitating entry into host cells. Viral-cell membrane fusion releases viral particles into the cell, where viral RNA is reverse-transcribed into DNA. In the cytoplasm, incomplete proviral DNA of HIV is recognized by the HIN-200 family of inflammasomes AIM2 and IFI6 via the HIN domain, leading to ASC recruitment followed by caspase-1 activation and inflammasome assembly. This process activates STING and caspase 1. STING activation triggers cell signaling pathways like NF-κB and MAP3K. HIV viral proteins and ssRNA induce ROS production, activating NLR family inflammasomes such as NLRP1 and NLRP3, which recruit ASC and assemble inflammasomes, ultimately activating caspase-1. Caspase-1 cleaves pro-IL-1β and pro-IL-18 into their mature forms. Additionally, GSDMs are activated by caspase-1 cleavage, forming cell membrane pores that trigger pyroptosis and facilitate IL-1β/IL-18 secretion. Abbreviations: Pyrin domain (PYD); adaptor molecule apoptosis-associated speck-like protein containing a CARD (ASC); Caspase recruitment domain (CARD); function-to-find domain (FIIND); Leucine-rich repeat (LRR); nucleotide-binding and oligomerization domain (NBD); stimulator of interferon genes (STING); Gasdermin (GSDM).

NLRP1 (NOD-like receptor containing a pyrin domain 1) was the first discovered PRR with the capacity to form the inflammasome complex. It possesses a unique domain architecture consisting of an N-terminal pyrin domain, a central NACHT and LRR, and a C-terminal CARD domain. Notably, NLRP1 also harbors a function-to-find domain (FIIND) that is not present in the other NLRP family members. This unique domain architecture of NLRP1 empowers it to directly recruit pro-caspase-1 through its CARD domain, bypassing the need for the adaptor molecule ASC. However, NLRP1 retains the ability to utilize ASC for the proteolytic activation and autoproteolysis of caspase 1 (Figure 2). NLRP1 activation leads to IL1β/IL18 processing, and pyroptosis using and apoptosis via caspase-2 and -9 [15]. A positive correlation exists between the increased expression of NLRP1, IL-1β, and IL-18 in the gut-associated lymphoid tissue (GALT) and peripheral blood, potentially contributing to the depletion of CD4+ T cells and high viral load, the hallmarks of rapid HIV progression [98]. NLPR1’s shared activation pathways with CARD8, an HIV-1 protease sensor, suggest it as a potential candidate for detecting HIV-1 infection via HIV-1 protease-mediated inflammasome assembly and triggering cell death [99]. NLRP1 can act as a viral “tripwire” by undergoing protease-mediated cleavage, triggering structural change and inflammasome activation independent of ligand binding [100]. In humanized mice, the elevated levels of NLRP1 in the lymph nodes suggest an early host inflammasome activation response to HIV-1 infection [101]. Despite being the prominent inflammasome in barrier cells like epithelial cells, deciphering NLRP1’s role in HIV infection remains challenging due to both the substantial divergence between human and murine NLRP1, limiting the applicability of disease models, and the current lack of specific NLRP1 agonists and antagonists to dissect its function in inflammation [102].

The NLRP3 inflammasome has been extensively studied among these inflammasomes, primarily because of its protective function in host defense against various infections and its involvement in several inflammatory disorders. NLRP3 requires exogenous activators to interact with ASC, facilitate the binding of ASC to CARD and the subsequent maturation of caspase 1, which then cleaves proinflammatory cytokines into their active forms. An investigation into single nucleotide polymorphisms (SNPs) within NLRP1, NLRP3, NLRC4, CARD8, CASP1, and IL1B genes revealed a significant association between two SNPs residing in NLRP3 and IL1B with increased susceptibility to HIV-1 infection [103,104]. Monocyte-derived dendritic cells (MDDCs) from PWH exhibited a dampened NLRP3 inflammasome response to HIV-1 compared to healthy donors (HDs), suggesting the potential exhaustion of this pathway during chronic infection. Conversely, HD MDDCs displayed a robust initial NLRP3 upregulation, highlighting its potential role in the early immune response against HIV-1 [105]. In addition, another evidence showed that NLRP3 proteins are modulated through post-translational modifications such as ubiquitination during HIV entry into the cells [106]. When the HIV envelope binds to its host cell receptor, it recruits the E3 ubiquitin ligase CBL to NLRP3, ultimately leading to NLRP3 degradation, thereby overcoming NLRP3 resistance in HIV-infected cells.

HIV-1 infection prompts inflammasome activation in microglia, leading to the release of IL-1β, which could contribute to inflammation and neuronal death, potentially leading to complex neurobehavioral deficits [107]. HIV Tat, cocaine, and cART can activate NLRP3 inflammasome signaling, leading to the generation of proinflammatory cytokines in microglial cells and iTat mice, potentially contributing to the dysregulated neuroinflammation observed in NeuroHIV in PWH on cART with cocaine addiction [108]. HIV-1 infection induces pro-IL-1β production in monocytes by activating TLR8 and subsequently activates caspase-1 through the NLRP3 inflammasome, leading to the cleavage of pro-IL-1β into its bioactive form, IL-1β, underscoring the essential roles of both TLR8 and NLRP3 in HIV-1-induced inflammation. Moreover, HIV infection in macrophages and peripheral blood mononuclear cells (PBMCs) from PWH showed increased expression levels of NLRP3 inflammasome components and downstream cytokines (including caspase-1, IL-1β, and IL-18), which correlated with various genes linked to cardiovascular disease. This study suggests that HIV infection may exacerbate the risk of cardiovascular disease in PWH by activating NLRP3 [109].

IFI16 (IFN-γ-inducible protein-16) is a nuclear pathogen sensor that contains one N-terminal PYD domain and one C-terminal HIN-200 domain, separated by an inter-domain linker region [110,111]. When IFI16 senses the foreign DNA, it recruits ASC to induce the activation of caspase-1 and inflammasome. After infection, the expression of IFI16 increases significantly but decreases during cART and it is positively associated with viral count [112]. IFI16 was shown to be sensing dsDNA but not RNA: DNA duplexes during the replication cycle of HIV in macrophages and controls the early HIV infection by hampering HIV-1 transduction and replication [27]. Sensing HIV DNA produced during reverse transcriptions by IFI16 induces a STING-dependent ISD pathway to trigger type I and III IFN responses [113]. IFI16 has been shown to play a crucial role in activating caspase-1 within inflammasomes, leading to pyroptosis in CD4+ T cells during HIV infection. Consequently, instead of protecting the host, the innate response driven by IFI16 results in debilitating CD4+ T-cell depletion, which is a key factor in the progression to AIDS in people living with HIV [114]. Additionally, another study identified a negative correlation between IFI16 expression and CD4+ T cell levels in cART-naive patients [28]. Studies in non-human primates have also shown that SIV-infected animals exhibit reduced CD4+ T cell counts alongside the elevated levels of IFI16, caspase-1, and IL-1β, with the decrease in CD4 count attributed to pyroptosis [115]. IFI16 is also capable of HIV restriction by suppressing host transcription factor Sp1 in CD4+ T cells and macrophages [116].

Like IFI16, AIM2, another member of the AIM2-like receptor family, functions as a sensor by detecting cytoplasmic and nuclear DNA from pathogens and damaged cells, thereby triggering an inflammatory response [117]. While IFI16 recognizes intracellular HIV single-stranded DNA (ssDNA), AIM2 senses and binds to double-stranded DNA. Subsequently, it recruits ASC to activate caspase 1, initiating acute inflammation and pyroptosis [118]. AIM2-mediated inflammasomes are initiated through the generation of reactive oxygen species (ROS) and the transcriptional activation of IL-1β when HIV-infected macrophages are exposed to cocaine, ultimately resulting in caspase 1-mediated apoptosis [119]. In PWH, particularly those with higher viral loads, a decrease in high-density lipoprotein (HDL) levels is associated with the upregulation of AIM2, NLRP3, ASC, IL-1β, and IL-18. This observation suggests that an increased activation of inflammasomes may lead to lower HDL levels, thereby reducing its anti-inflammatory effect, which could potentially contribute to the increased pyroptosis of CD4+ T cells [120].

Caspase recruitment domain-containing protein 8 (CARD8) is the only other protein besides NLRP1 that possesses a FIIND domain. However, unlike NLRP1, CARD8 lacks N-terminal pyrin domain (PYD)—NACHT (NAIP, CIITA, HET-E, and TP-1) and LRRs [121]. CARD8 and NLRP1 inflammasomes, despite lacking identical N-termini, share a similar activation pathway involving microbial protease cleavage and the proteasome-dependent release of a C-terminal fragment for inflammasome assembly and caspase-1 activation. CARD8 directly senses the activity of HIV protease, a viral enzyme, by being cleaved at a specific site within its unstructured N-terminus [99]. This cleavage activates CARD8, initiating a cascade of events that culminates in the assembly of the inflammasome complex and the subsequent inflammasome-mediated pyroptosis of HIV-1-infected cells. Recent studies by the same group suggest that a loss-of-function mutation in CARD8, rendering it uncleavable by SIV in sooty mangabeys, might explain why they do not experience CD4+ T cell depletion after infection. This implies that the HIV/SIV activation of CARD8 could play an indispensable role in CD4+ T cell depletion [122].

NLRC4 lacks a PYD domain, and inflammasome assembly is formed by recruiting ASC directly through their CARD like NLRP1. Patients undergoing cART displayed elevated expression levels of various inflammasome components (NLRP1, NLRP3, NLRC4, AIM2, ASC, and caspase 1), which were correlated with triglyceride and VLDL levels. This suggests that inflammasome activation may contribute to elevated cardiovascular risk in individuals on ART. Research conducted on primary monocyte-derived macrophages (MDMs) revealed that IL-1β induced by HIV primarily relies on the NLRP3 inflammasome for regulation, whereas IL-18 production is primarily governed by the NAIP/NLRC4 inflammasome [123]. HIV-1 viroporin (pore-forming viral protein) glycoprotein 41 (gp41) activates the NAIP/NLRC4 by binding directly with NAIP and triggers mainly IL-18 production.

### 5.2. Inflammasomes and Inflammaging

In attempts to identify the key drivers and mechanisms of inflammaging, recent research highlights the critical role of inflammasomes in the sustained activation of inflammatory pathways in elderly individuals. These cellular complexes trigger in response to pathogens like HIV, exogenous factors such as certain cART drugs, or immune dysfunction due to aging. Studies have revealed a potential link between the persistently elevated expression of NLRC4 and NLRC5 inflammasome components and a cluster of age-related pathologies [124]. Individuals with this expression pattern exhibit increased blood pressure, arterial stiffness, chronic inflammatory cytokine levels, metabolic dysfunction, oxidative stress, and a shortened lifespan. Notably, the research suggests a potential mechanism: reactive oxygen species (ROS) can activate NLRC4. This suggests that increased oxidative stress, a hallmark of aging, might trigger higher tRNA degradation, consequently upregulating NLRC4 gene expression. This persistent oxidative stress triggers an inflammatory response through inflammasome proteins. These proteins induce the production of IL-1β, IL-6, and IL-23 by dendritic cells and IFN-γ and IL-17 by T cells, which, in turn, infiltrate the kidneys and vessels, causing sustained hypertension. When overactivated in mice, the NLRC4 inflammasome triggers an inflammasome-dependent cell death pathway, resulting in either pyroptosis or apoptosis, leading to tissue/organ damage and lethality in animals [125].

In the aged brain, research indicates an age-dependent association between NLRP1, ASC, and caspase-8, forming a complex that may contribute to neuroinflammation and neurodegenerative diseases [126]. Elevated levels of NLRP1, ASC, caspase-1, and caspase-8 were observed in the cortex of aged mice, and treatment with anti-ASC decreased the expression of these proteins, resulting in lower levels of IL-1β. This finding underscores the potential link between increased inflammatory activity and age-related changes in brain function. The studies of hippocampal tissue from aged rats, a brain region vital for memory, revealed a link between the elevated levels of NLRP1 inflammasome components, including caspase-1, caspase-11, the P2X7 receptor, pannexin-1, and XIAP, and cognitive decline [127]. However, treatment with the gout anti-inflammatory drug probenecid reduced inflammasome activity, resulting in lower levels of IL-1β and IL-18, and improved spatial learning performance in aged rats. Older mice show elevated senescence-associated activity of β-galactosidase (β-gal), along with increased levels of ROS and IL-1β, NLRP1, ASC, caspase-1, NOX2, p47phox, and p22phox in the cortex and hippocampus. The involvement of NOX2-NLRP1 inflammasome signaling in aging-related neuronal damage suggests its potential as a target for modulating brain aging [128]. Another study showed that the NLRP1/CXCL1/CXCR2-BNDF signaling pathway contributes to the effect of age on chronic stress-induced depressive-like behavior in aged mice and Nlrp1a knockdown reduced the levels of proinflammatory cytokines [129]. HIV proteins can induce high NLRP3 expression in astrocytes and brain tissue, potentially contributing to memory and motor decline in patients with HIV. These effects may be similar to the neuropathological changes observed during aging, including increased astrocyte activation and altered synaptic plasticity. The presence of HIV-1 Tat protein in PWH undergoing cART treatment might therefore increase the risk of accelerated brain aging in this population [15]. These results demonstrate that inflammasomes play a critical role in regulating the inflammaging response in the central nervous system, not only in microglia but also in astrocytes and neurons.

Studies involving PWH have identified a positive correlation between the expression of NLRP3 and IL-1β genes with the progression of neurocognitive impairment [130]. NLRP3 inflammasome increased in the hippocampus of natural aging rats which contributed to the impairment of synaptic plasticity and cognition [131]. Studies investigating inflammasome function found that inhibiting Nlrp3 inflammasome activation yielded multiple benefits in aging mice. These included protection from age-related increases in innate immune activation, improved glycemic control, reduced bone loss, and preservation of thymus function. Interestingly, only Nlrp3 inflammasome inhibition improved cognitive function and motor performance in aged mice, suggesting a specific role for Nlrp3 in these age-related declines [132].

Autophagy, a cellular recycling process, normally eliminates damaged components like dysfunctional mitochondria, and can suppress NLRP3 inflammasome activation and promote cellular health. Proteins associated with autophagy have been found to inhibit the components of the NLRP3 inflammasome, including ASC and NLRP3 [133,134]. Furthermore, the inhibition of autophagy associated with TLR3 or TLR4 signaling leads to the activation of the NLRP3 inflammasome and the secretion of IL-1β and IL-18 [135]. However, chronic HIV infection can trigger inflammasome activation, leading to a proinflammatory environment that impairs autophagy, potentially accelerating the aging process [130,136]. Another study suggests that inhibiting NLRP1 inflammasome activation corrects AMPK/mTOR-mediated autophagy dysfunction, resulting in improved learning and memory [137]. Thus, a feedback loop exists between inflammasomes and autophagy, wherein autophagy components can impair inflammasome proteins, while inflammation itself hampers autophagy’s efficiency. Age-associated decline in autophagy and the subsequent defects in mitochondrial uptake and degradation lead to heightened inflammasome activity mediated by reactive oxygen species (ROS) [138,139]. This dysregulation worsens with age and contributes to chronic inflammation and the pathogenesis of various age-related diseases.

### 5.3. Inflammasomes as Therapeutic Targets

**Figure 3:**
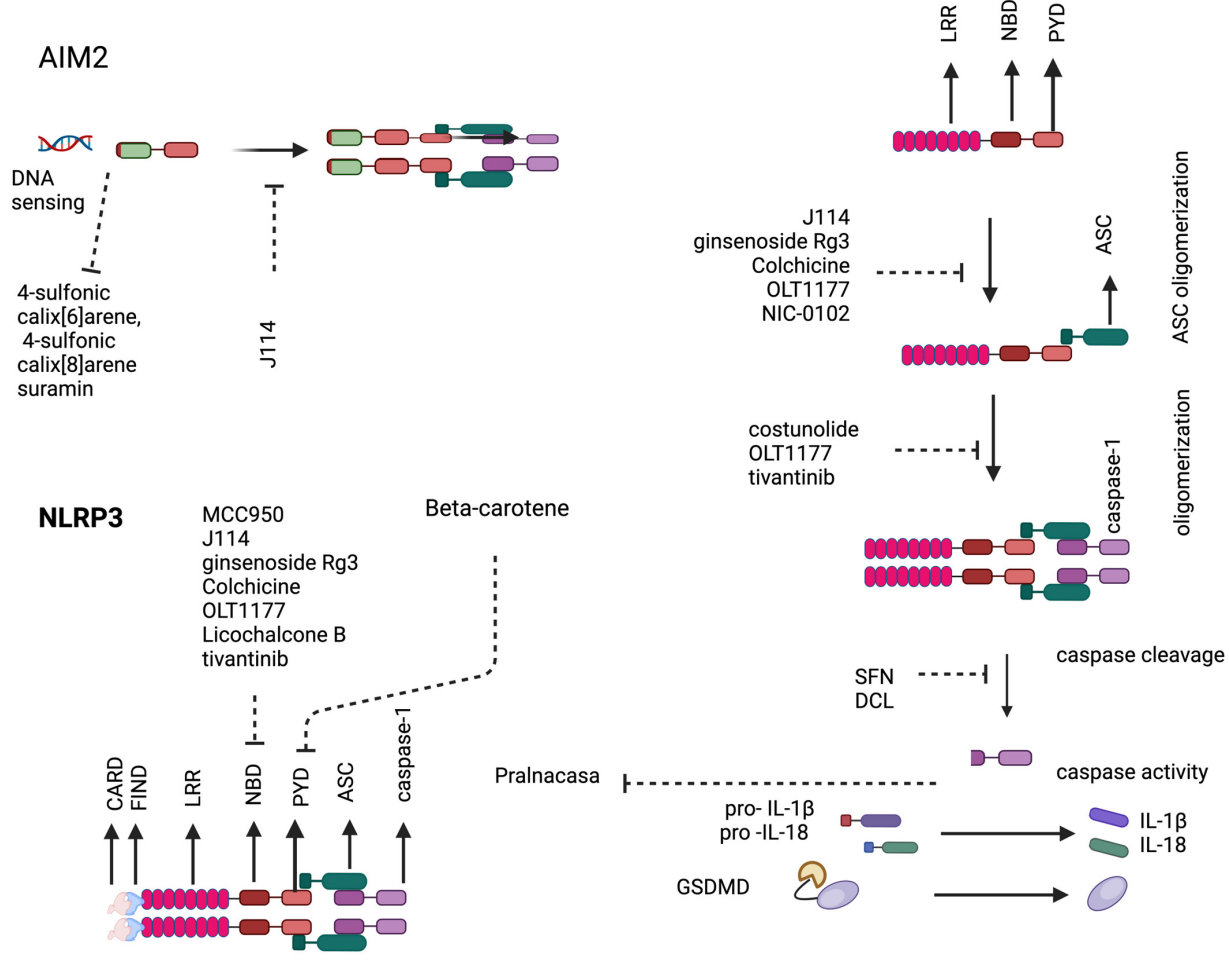
Inhibitors of inflammasome pathway. Various inhibitors target different components of the inflammasome pathways, including NLRP3, AIM2, ASC, caspase-1, IL-1β, pro-IL-18, and Gasdermin-D (GSDMD). These inhibitors prevent inflammasome activation, subsequently inhibiting pyroptosis and the release of proinflammatory cytokines.

Given the role of inflammasomes in age-related diseases and HIV infection, the potential of inflammasomes as therapeutic targets for inflammatory diseases in older individuals and PWH is promising (Figure 3). Recent studies have identified several compounds with the potential to modulate dysfunctional inflammasome pathways. The compound J114, also referred to as N-(3-hydroxyphenyl)-2-(1H-indol-6-yl) acetamide, exhibited the inhibition of caspase-1 activation and IL-1β release by directly disturbing the interaction of NLRP3 or AIM2 with the adaptor protein ASC and inhibited ASC oligomerization in human THP-1 macrophages [140]. This compound prevented acute corneal inflammation and cell injury by inhibiting the noncanonical pyroptosis signaling pathway in mice [141]. Compounds like 4-sulfonic calix [6]arene, 4-sulfonic calix [8]arene, and suramin have been identified as the inhibitors of dsDNA-triggered inflammatory responses mediated by the AIM2 inflammasome. They target the dsDNA-binding site located in the HIN domain of AIM2, thereby blocking dsDNA binding and hindering AIM2 inflammasome formation. At higher concentrations, 4-sulfonic calixarenes can also disrupt the activity of other dsDNA sensors like cGAS-STING and TLR9. Additionally, suramin has been shown to inhibit the processing of caspase-1 and IL-1β by blocking AIM2-dependent ASC oligomerization [142].

The naturally occurring isothiocyanate sulforaphane, found in broccoli sprout extracts and consumed as a dietary supplement, inhibits caspase-1 autoproteolytic activation and IL-1β maturation and secretion downstream of the NLRP1, NLRP3, NAIP5–NLRC4, and AIM2 inflammasomes [143]. Glycyrrhiza, commonly used in traditional Chinese medicine, contains various active compounds like Licochalcone A, echinatin, and Glycyrrhizin, which have been found to inhibit the activity of the NLRP3 inflammasome [144]. Another traditional Chinese medicinal herb Saussurea lappa contains the bioactive compound costunolide which covalently binds to cysteine 598 within the NLRP3 inflammasome’s NACHT domain, disrupting its ATPase activity and hindering inflammasome assembly [145]. Saussurea lappa also contains dehydrocostus lactone (DCL), which suppresses NLRP3 inflammasome activation in primary mouse macrophages and human peripheral blood mononuclear cells [146]. DCL inhibits caspase-1 activation and IL-1β production, thereby exerting an anti-inflammatory effect by targeting the NLRP3 pathway. Panax ginseng’s major constituent, ginsenoside Rg3, disrupts interactions between NEK7 and NLRP3, as well as NLRP3 and ASC. This hinders ASC oligomerization, thereby reducing IL-1β secretion and caspase-1 activation, ultimately inhibiting the NLRP3 inflammasome [147].

OLT1177, an orally active β-sulfonyl nitrile molecule, effectively inhibits both the canonical and noncanonical pathways of the NLRP3 inflammasome, thereby reducing the secretion of IL-1β and IL-18. Additionally, OLT1177 decreases the interactions between NLRP3 and ASC, as well as NLRP3 and caspase-1, by impeding the oligomerization of the NLRP3 inflammasome and subsequent caspase-1 activity [148]. Colchicine, a widely used gout medication with anti-inflammatory properties, disrupts the NLRP3 inflammasome pathway through a multi-faceted mechanism. It disrupts microtubule function, thereby hindering the critical interaction between ASC and NLRP3, which is essential for inflammasome assembly [149]. Additionally, colchicine may suppress caspase-1 activation by reducing its mRNA transcript levels, leading to the decreased protein expression of caspase-1 [150]. Pralnacasan, an orally administered small molecule inhibitor of the interleukin-1β converting enzyme (ICE), demonstrates the potential for treating osteoarthritis in mice models by reducing caspase activity [151].

In preclinical trials, MCC950, a small-molecule NLRP3 pathway inhibitor, was found to directly interact with the NACHT domain of NLRP3. This interaction prevents ATP hydrolysis, thereby inhibiting NLRP3 activation and the formation of inflammasomes [152]. Beta-carotene, a plant-derived provitamin A, directly binds to the PYD of NLRP3, thereby inhibiting its interaction with ASC and suppressing NLRP3 activation [153]. The orally bioavailable proteasome inhibitor NIC-0102 disturbs the NLRP3-ASC interaction, preventing ASC oligomerization and ultimately leading to the inhibition of NLRP3 inflammasome activation and the production of pro-IL-1β [154]. The anticancer agent tivantinib is an inhibitor of NLRP3 which acts by interfering with NLRP3 ATPase activity and subsequent inflammasome complex assembly [155]. There are several other compounds that are under investigation in preclinical and clinical studies [156]. While these studies offer promising prospects for the development of drugs targeting inflammatory responses mediated by inflammasomes, their clinical efficacy and long-term safety remain largely unproven and await validation through randomized controlled trials involving human subjects. Additionally, further research is needed to assess their potential therapeutic benefits, optimal dosages, potential adverse effects, and potential drug interaction with cART in human populations.

## 6. Conclusions

Inflammasomes are an important arm of the innate immune system, sensing pathogens and responding with the synthesis and release of immune defense molecules. However, the dysregulation of the inflammasome pathway leads to immune senescence and inflammaging in both treated and untreated PWH as well as the elderly population. Moreover, uncontrolled inflammasome activation is closely connected to the onset and progression of diverse age-related comorbidities, including cardiovascular diseases, arthritis, type 2 diabetes, hypertension, metabolic, and neurodegenerative diseases.

As our understanding of inflammasome assembly solidifies, future research will delve deeper into the structural intricacies of ligand–sensor binding, interactions between various inflammasome sensors, and the formation of noncanonical inflammasomes. Additionally, exploring the functional outcomes of inflammasome activation on other cellular processes, such as intestinal and mucosal barrier function, transcription, alternative cell death pathways, and more, presents enticing prospects. Deciphering the intricate details of inflammasome interactions with other cellular proteins and ligand–sensor interactions during HIV/aging will be a crucial aspect to focus on. Targeting these pivotal inflammasome pathways carries immense therapeutic potential, thereby paving the way for novel treatments and preventive strategies for a broad spectrum of diseases associated with HIV/aging.

Ultimately, our comprehension of the involvement of inflammasome dysregulation in aging and HIV infection is hindered by the restricted conservation of inflammasome proteins across human and rodent species. Therefore, advancing inflammasome research necessitates the creation of more clinically relevant models that can bridge the gap between preclinical studies and human therapeutic applications. It is essential to broaden inflammasome investigation by leveraging non-human primates, humanized mice, organ-on-a-chip technology, or other innovative methodologies that offer a more accurate representation of the human inflammasome physiology.
